# Simple Growth Patterns Can Create Complex Trajectories for the Ontogeny of Constitutive Chemical Defences in Seaweeds

**DOI:** 10.1371/journal.pone.0086893

**Published:** 2014-01-30

**Authors:** Nicholas A. Paul, Carl Johan Svensson, Rocky de Nys, Peter D. Steinberg

**Affiliations:** 1 School of Marine and Tropical Biology, James Cook University, Townsville, Queensland, Australia; 2 Department of Marine Ecology, Göteborg University, Göteborg, Sweden; 3 School of Biological, Earth and Environmental Sciences, and Centre for Marine Bio-Innovation, University of New South Wales, Sydney, Australia; University of Sydney, Australia

## Abstract

All of the theory and most of the data on the ecology and evolution of chemical defences derive from terrestrial plants, which have considerable capacity for internal movement of resources. In contrast, most macroalgae – seaweeds – have no or very limited capacity for resource translocation, meaning that trade-offs between growth and defence, for example, should be localised rather than systemic. This may change the predictions of chemical defence theories for seaweeds. We developed a model that mimicked the simple growth pattern of the red seaweed *Asparagopsis armata* which is composed of repeating clusters of somatic cells and cells which contain deterrent secondary chemicals (gland cells). To do this we created a distinct growth curve for the somatic cells and another for the gland cells using empirical data. The somatic growth function was linked to the growth function for defence via differential equations modelling, which effectively generated a trade-off between growth and defence as these neighbouring cells develop. By treating growth and defence as separate functions we were also able to model a trade-off in growth of 2–3% under most circumstances. However, we found contrasting evidence for this trade-off in the empirical relationships between growth and defence, depending on the light level under which the alga was cultured. After developing a model that incorporated both branching and cell division rates, we formally demonstrated that positive correlations between growth and defence are predicted in many circumstances and also that allocation costs, if they exist, will be constrained by the intrinsic growth patterns of the seaweed. Growth patterns could therefore explain contrasting evidence for cost of constitutive chemical defence in many studies, highlighting the need to consider the fundamental biology and ontogeny of organisms when assessing the allocation theories for defence.

## Introduction

A cornerstone for explanations of variation in plant chemical defences is that there is a cost of defence, such that growth, reproduction or other plant properties are constrained as a result of resources being diverted towards the synthesis of secondary metabolites and structures for their storage and transport [1]–[4]. Superficially, such costs of chemical defences imply that levels of defences and quantitative variation in other plant traits, such as growth, should be negatively correlated. However, it is now clear from empirical studies of cost that this is not always the case [5]. The absence of correlative evidence for cost could indicate that growth and defence concentrations are not tightly coupled [3], [6], [7]. A lack of correlative evidence may also be due to the fact that growth is often only measured for a single ontogenetic stage rather than the entire developmental trajectory [8], [9]. This is potentially problematic, as the intrinsic growth patterns of a particular stage could impose its own constraints, independent of any trade-offs with defence [10]. Consequently, ontogeny is now seen as a significant constraint for understanding trade-offs between chemical defences and other traits in terrestrial plants [5], [8].

Trade-offs in seaweeds (marine macroalgae) may be less constrained by ontogeny than terrestrial plants, as many seaweeds are short-lived ephemerals with fast growth rates, and often lack a systemic circulatory system. Source-sink relationships are therefore more localised than vascular plants [11]. Seaweeds tend to have highly variable concentrations of secondary metabolites both within and among individuals for both constitutive and inducible defences [12]–[18]. In a number of instances this variation has been related to variation in resources such as light or nutrients [19]–[21], as theories of resources allocation would predict. A unifying model for resource allocation is attractive and should apply to both constitutive and inducible defences, to plants with fundamentally different allelochemicals and morphologies, and to both terrestrial and marine systems [22], [23]. However, as with higher plants, the evidence for cost of seaweed chemical defences can be correlative and equivocal [24] or even conflicting [12]. These results are challenging to reconcile and suggests that allocation costs – if present – may often be marginal, irrespective of whether the chemical defences are constitutive or inducible.

A model that formalised potential trade-offs between growth and defence in seaweeds could potentially aid in interpreting the mixed results from empirical studies. We believe that developing this model is particularly important because multiple approaches exist for investigating trade-offs between growth and defence, from energetic calculations of the metabolic costs of producing and storing metabolites [25], [26] through to fitness costs or ecological costs associated with lost opportunity [3], [4], [7], and each has their own limitations. A blend of complementary approaches is both possible and a benefit to a mature field, such as terrestrial plant defences, where metabolic pathways are well-established and in many cases genetically modified plants are used to test and build hypotheses for both inducible and constitutive defences [6]. However, significant knowledge gaps remain for seaweed research in each of these areas, and these gaps are compounded by the diversity of taxa (brown, red and green algae) and types of defences (constitutive, inducible and activated [17], [22], [23]) that fall under the broad banner of “seaweed chemical defence”. As a first step any model for seaweeds should attempt to accommodate the intrinsic differences between terrestrial plants and seaweeds, such as the limited translocation of resources in many seaweeds (although see [11], [27]). Secondly, the model can initially be used to address constitutive chemical defences in seaweeds, as these are more difficult to study empirically as constitutive cannot be quickly manipulated. The benefit of modelling allocation to constitutive defence in seaweeds is that it also allows us to begin with realistic examples of organisms with very simple growth patterns, such as filamentous algae, as a base for seaweeds with more complex body plans and greater ability to translocate resources. Filamentous algae are very diverse, they grow rapidly and have relatively short life histories, are typically small as mature adults, and can also be chemically defended [14], [28], [29]. Furthermore, in some species, chemical defences are sequestered within specialised ‘gland’ cells [30]. This means that the simple growth patterns of filamentous algae can be linked to the production and accumulation of constitutive secondary metabolites in specific cells.

We explored the relationships between growth and constitutive defence in the filamentous red alga *Asparagopsis armata* using both empirical and modelling techniques. We firstly manipulated growth rates and whole-individual concentrations of chemical defences by changing light availability in culture. Our goal was to determine whether differing light availability resulted in empirical correlations of different strength or sign [3], [31]. We then characterised the growth patterns of *A. armata* and used these cellular data as the basis for a set of differential equations that modelled the relationship between growth and constitutive defence. Light availability was also mimicked in the model through changes in branching and cell division rates, enabling us to generate modelled individuals with different ontogenetic trajectories of cell development and therefore a different history of investment in somatic or gland cells. This allowed us to directly compare the empirical correlations between growth and defence of whole individuals with the predictions from the model using the parameters derived from the cellular data.

## Materials and Methods

### Study Organism

The red alga *Asparagopsis armata* Harvey (Bonnemaisoniaceae) has a life cycle with an alternation of generations between foliose, dioecious gametophytes and a filamentous sporophyte (tetrasporophyte). The natural products chemistry has been well described [28], [32] and both the tetrasporophyte and gametophyte contain high and effective levels of halogenated secondary metabolites [28], [33]. In this study we focus on the filamentous tetrasporophyte. This alga produces simple brominated hydrocarbons such as bromoform and dibromoacetic acid [32], which are active against multiple herbivores [29], [33] and inhibit marine bacteria [28]. Individuals used in this study were collected from two shallow subtidal sites, Bare Island (33° 59′ 38″ S, 151° 14′ 00″ E) and Long Bay (33° 59′ 10″ S, 151° 14′ 15″ E), Sydney, Australia (with permission by New South Wales Fisheries) and maintained in culture under constant temperature (19°C) and light (at ∼40 µmol photons m^−2^ s^−1^) on a 16∶8 light:dark cycle. *A. armata* is readily propagated from excised filaments and its cellular components and growth pattern can be viewed easily *in vivo* with light microscopy [30], [34].

**Figure 1:**
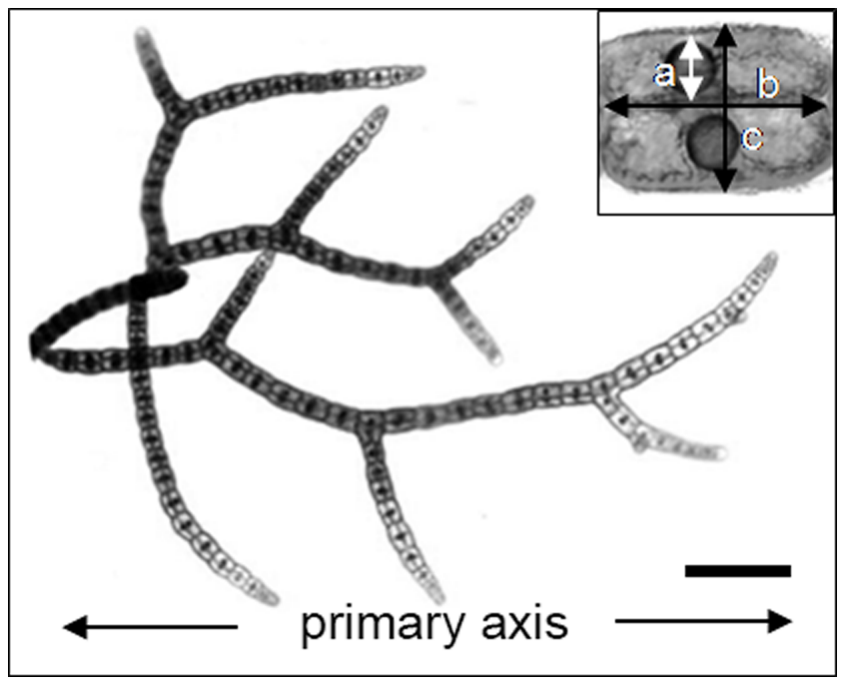
Habit of the filamentous tetrasporophyte of *Asparagopsis armata*. Outside of branching events, cell division only occurs at the single apical cell on each axis. Gland cells are first observed approximately 3–5 cells from the apical cell. Inset: the repeating unit of cellular growth is a tier comprised of 7 cells. Each cell tier (a cylinder of length “b” and diameter “c”) consists of 3 pericentral cells surrounding a single axial cell, and each pericentral cell contains a single refractile gland cell (a sphere of diameter “a”). Scale bar, 200 µm.

The filaments have apical growth and regularly branch. The filamentous axis is a repeating linear arrangement of cell clusters, each comprised of an axial cell and three surrounding pericentral cells with associated gland cells (Fig. 1 inset). We use the term “cell tier” to refer to these repeating clusters of cells (1 axial, 3 pericentral and 3 gland cells, giving a total of 7 cells in each tier). The gland cell is a dense structure that occupies space within the pericentral cell (Fig. 1 inset: 2 pericentral cells and associated gland cells; and see [30]). We have previously demonstrated that the production of chemical defence is directly linked to the presence of a large refractile inclusion in the gland cell, for when *A. armata* is grown in the absence of bromine the halogenated metabolites are no longer detected and the inclusion is absent [28], [30]. Both small and large gland cells have similar consistency and distributions of refractile contents based on observations with transmission electronic microscopy [30].

### Empirical Correlations of Growth and Defence

We used light availability to manipulate growth rates of *A. armata* and correlated the resulting variation in growth with variation in metabolite concentrations of whole individuals. The two light treatments provided photosynthetically active radiation at 10 µmol photons m^−2^ s^−1^ and 35 µmol photons m^−2^ s^−1^. We refer to these treatments as low and moderate light, as both growth and bromoform concentration steadily increased in a preliminary assay with increased light from 4 µmol photons m^−2^ s^−1 ^up to 60 µmol photons m^−2^ s^−1^. Light in seawater is generally considered limiting for sub-tidal algae below 100 µmol photons m^−2^ s^−1^ [35] and this has been demonstrated empirically for *A. armata* at 19°C, for which photosynthetic capacity increased linearly with light availability up to 100 µmol photons m^−2^ s^−1^ [36]. The light treatments used in our study would be relevant to shaded habitats, such as those beneath canopies of larger seaweeds where the tetrasporophytes of *A. armata* can often be found.

A total of 15 individuals were collected and three small (10–15 cell) apical sections were excised from each individual for each light treatment. Individual lines (families) were maintained and mean values of the clones were used in the correlations between growth and defence. Algae were cultured in sterile seawater ½ strength Provasoli Enrichment Solution for 4 weeks [37]. The culture medium was changed weekly. Due to their small size (<0.1 mg fresh weight), growth was quantified as changes in area using digital images of prostrate samples (beneath a coverslip) obtained with a stereomicroscope. Growth was measured after 4 weeks and the levels of the major halogenated metabolites in each replicate analysed by gas chromatography – mass spectrometry [28]. The four major metabolites are bromoform, dibromoacetic acid (DBA), bromochloroacetic acid (BCA), dibromochloromethane, which together accumulate to high concentrations (on average 25 µg mg^−1^ or 2.5% DW [28]). Metabolites are reported as mass per unit area (µg mm^−2^).

**Figure 2:**
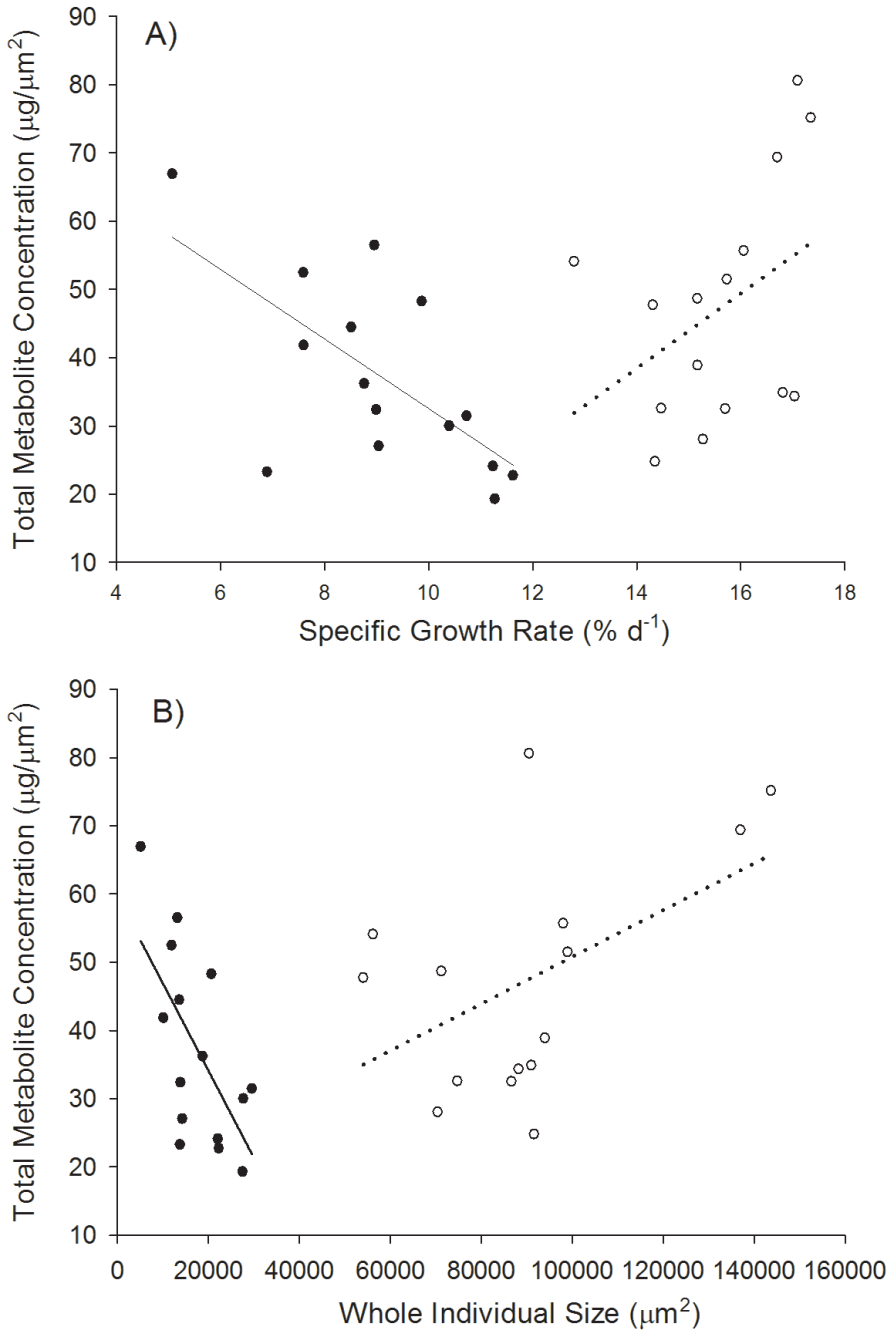
Empirical correlations between total metabolite concentration and growth rate (A) and individual size (B) for 15 families of clones under both low light (solid circles) and moderate light (no fill). Solid lines show significant negative correlations (*P*<0.05), dotted lines show positive trends (*P* = 0.13 A, *P* = 0.07 B).

Correlations were calculated between growth rate and metabolite concentrations using the means (*N* = 2−3) of each family (*N* = 15) for each light level (as per [12], [31]). Such family level correlations reflect underlying genotypic correlations [38] and the gradient of this correlation is considered an indicator of the effect size for the trade-off between growth and defence [3]. We used specific growth rate (SGR, % d^−1^), which is the ln-transformation of the final size over the initial size divided by the number of days in culture [ln(final size/initial size)/28*100]. We also assessed the correlations between final size and concentration of metabolites to make comparisons with the outputs of the modelling which was based on the size of individuals (see later). The correlation co-efficients for each light level were re-sampled and we report the *P* and *r* values from the resampling in the results (Fig. 2a & b: 1,000 simulations, Statistics101 Resampling Simulator). Potential bias from using size simultaneously on the x-axis and in the denominator of the ratio on the y-axis (Fig 2b: see [39]) was assessed by producing a corrected gradient for each correlation using randomly generated data truncated to the range of x- and y-values for each light level. The correlation at low light for the corrected data remained negative (*r* = −0.200: and see Results).

### Cellular Patterns of Resource Allocation

One of our fundamental assumptions for the cost of constitutive chemical defence in these algae is that any trade-offs between defences and growth will only occur at the localised level of the cell tier. Thus a measure of allocation in chemical defence at the cellular scale was necessary for developing a growth model for trade-offs in resource allocation in *A. armata*. It was not possible to measure the metabolite concentrations of individual cells or clusters of cells in small filaments with the analytical techniques used above for larger thalli. We therefore used within-individual comparisons of cell sizes as a separate measure of allocation to chemical defences. The critical assumption of the model is that the size of the gland cells relative to the cell tier represents a surrogate measure of allocation to chemical defence. This assumption is based on a previously demonstrated relationship between the concentration of halogenated metabolites (presence and absence) and the size (presence and absence) of the large vesicle in the gland cells of *Asparagopsis* ([28], [30], see also related information in “Study Organism”). Importantly, this measure of defence also captured all associated metabolites (pre-cursors and minor compounds included) and the structures (gland cells) that contain them, which themselves have been argued to be as or more costly than the metabolites (e.g. [40]). Using this cellular proxy for chemical defence we were therefore able to quantify changes in allocation to defence along the filamentous growth axis, to our knowledge the first time it has been investigated at this scale, taking into account both the development stage of the cells and intrinsic growth patterns, such as branch frequency and cell division rates.

Three measures were needed to calculate the volumes of the cell tier and the gland cells (Fig. 1, inset): cell tier length and filament width at the centre of the cell tier were used to calculate cell tier (or total) volume (circular prism, inclusive of gland cells), and the diameter of the gland cell in the pericentral cell on the highest focal plane was used to estimate the combined gland cell volume (i.e. the volume of 3 spherical gland cells) for each cell tier.

Cellular measurements were made on a subsample of individuals used for the empirical correlations (above). Comparisons between algae (*N* = 7 families) cultured at 10 and 35 µmol photons m^−2^ s^−1^ were made in two ways. Firstly, the relative volume of the gland cells to the volume of cell tier was compared between apical and mature growth regions (*N* = 10 cell tiers per region) of the primary growth axis of each individual. Measurements were made for apical cells (young cells, 0–300 µm from the tip) and mature cells (older cells, 700–1000 µm from the tip). Mean values for each region were compared by mixed model ANOVA, with light (10 and 35 µE) and region (Apical and Mature) as fixed factors, and family (clones from *N* = 7 individuals) as a blocked factor in an unreplicated complete block design. Secondly, the relative size of the gland cell to the cell tier volumes (percentage) for each cell tier was measured from the apex to 1000 µm along the axis. These data defined the cellular growth parameters for the model (see following section).

### Modelling Growth and the Cost of Defence in *A. armata*

In this study we use differential equations modelling techniques to simulate the growth and development of *A. armata* (see [41] for a similar approach). The model mimics the cellular growth pattern of *A. armata* (see Fig. 1) and operates in discrete stages defined by cell division. Individuals begin as one cell tier and at each cell division a new cell tier is added to the previous, progressively producing a linear array of cell tiers. Individuals may branch at any desired frequency, which is determined by the average number of cells between branches (*b*). In this way the model is not defined by time or cell division rates but by the size of the individual.

The model quantifies the continuous growth of the cell tier (encompassing somatic growth) and the associated gland cells separately, treating them as two distinct functions (eqn.1 & eqn.2 below). As an individual grows, the relative size of gland cells to cell tier is calculated and represents the total relative allocation to chemical defence. Cell tier growth and gland cell growth are both size dependent (i.e. there is a maximum cell size, determined by empirical data: Table 1) and are described by sigmoid functions. The general expression for the change in gland cell size (*G*) in each cell division (*t*) is:
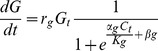
(1)where *r_g_* is the intrinsic growth of gland cells, *K*_g_ is the maximum size of the three gland cells collectively, and *α*_g_ and *β_g_* respectively determine the smoothness of the size function and the onset of size dependence (see Table 1 for additional model parameters derived from cellular data for low and moderate light individuals).

**Table 1:** Model parameters that describe the cellular growth patterns of *Asparagopsis armata*.

Symbol	Description	Light level	
		10 µE	35 µE
*S*_c_	Initial size cell tier (µm^3^)	1,300	1,400
*r*_c_	Cell tier maximum intrinsic growth rate	0.8	1
*K*_c_	Maximum cell tier size (µm^3^)	54,000	100,000
*b*	Average number of cells per branch	16±7	11±6
*α*_c_	Density function parameter	6	5
*β*_c_	Density function parameter	−0.05	−0.05
*S*_c_	Initial size gland cell (µm^3^)	15	35
*r*_g_	Gland cell maximum intrinsic growth rate	0.8	1
*K*_g_	Maximum gland cell size (µm^3^)	1,750	3,000
*α*_g_	Density function parameter	4	3.8
*β*_g_	Density function parameter	0.5	0.7

Parameters are derived from cellular data for individuals cultured under low (10 µE) and moderate (35 µE) light levels.

A cost of chemical defence in the model constrains the growth of the cell tier, as the growth of the cell tier is partially dependent on the relative size of the associated gland cells. Importantly the cost is expressed only in the isolated cell tier. This gives the following expression for the rate of change of cell tier size (*C*):
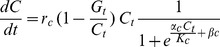
(2)where *r*_c_ is the intrinsic growth of the cell tier, and G_t_/C_t_ is the cost of defence. *α*_c_ and *β_c_* determine the smoothness of the size function and the onset of size dependence.

### Growth Patterns and Model Development

The model to this point replicates the cellular growth parameters of a single filament. As a final step we then parameterised the model with branch frequency and cell division rate, as both directly influence the growth pattern of the algae. Branch frequency in the apical region (# cells between branches) and the position of the first cell tier that contained a gland cell were determined from the earlier experiment where individuals were grown at two light levels (10 µE and 35 µE). We subsequently measured cell division rates under both light levels to provide inputs which model growth trajectories over time. This was done with six new individuals from one field site (Bare Island). Four apical portions were excised and acclimatised under 10 µE and 35 µE for 4 days. The number of new cells in the filament was quantified in the following 3 day period. The cell division rates (*N* = 6 families per light level) were compared by mixed model ANOVA with light as a fixed factor and family as a blocked factor.

The model was finally used to sum the cell tiers to the scale of whole individuals. By projecting the development forward by cell division, including branching patterns, we were able to monitor pooled (whole individual) changes in chemical defence relative to increases in size. We did this initially using mean values for branching and cell division and then subsequently manipulated growth patterns further using a range of empirical values, including interactions of branching and cell division rates under low and moderate light. To do this a coefficient of variation of 0.25 and 0.05 was set to represent low and high variance in cell division rate between individuals for low and moderate light, respectively (Table 1). Simulations also included random variation in branching rate based on the empirical data (see Results: “Scaling the model to whole individual”). For each of the two levels of variation in rate of cell division, we then randomly sampled 40 individuals modelled under low or moderate light and correlated growth and defence for each light level independently. This provided direct comparisons of the modelled outcomes with the empirical correlations of growth and defence.

## Results

### Empirical Correlations of Growth and Chemical Defence

Significant correlations between variation in growth rate and total metabolite concentration of the 15 families were observed for both low and moderate light levels but differed in sign (Fig. 2A). Under low light, total metabolite concentration negatively covaried with growth rate (*P* = 0.006, *r* = −0.659). A decrease in growth rate with increasing concentration of metabolites is usually taken as an indication of an allocation cost for secondary metabolite production. However, under moderate but still limiting light (35 µE), the pattern was reversed and there was a trend towards a positive correlation between growth rate and metabolite production (Fig. 2A: *P* = 0.129, *r* = 0.405). The mean growth rate of individuals for 10 µE was 9.1% d^−1^ (±0.47 SE) and for 35 µE was 15.6% d^−1^ (±0.33 SE). The correlation between size and total metabolite concentration was more pronounced than that for growth rate (Fig. 2B: *P* = 0.010, *r* = −0.646). Similar to the growth rate comparisons, there was a tendency towards a positive correlation between size and total metabolite concentration (*P* = 0.068, *r* = 0.492).

The two major metabolites in *A. armata* were bromoform (10 µE: 85.9% ±2.0 SE of the four evaluated; 35 µE: 79.5% ±2.0 SE) and dibromoacetic acid, DBA, (10 µE: 10.0% ±2.0 SE; 35 µE: 17.5% ±2.4 SE). The correlations between growth rate and specific metabolite concentrations varied in the strength and sign for the two major metabolites and also for the minor metabolites. Under low light, the concentrations of three metabolites negatively covaried with growth rate, including bromoform (*P* = 0.017, *r* = −0.605), dibromochloromethane (CHBr_2_Cl) (*P* = 0.049, *r* = −0.521) and dibromoacrylic acid (*P*<0.001, *r* = −0.835). DBA and bromochloroacetic acid (BCA) showed no significant correlations. When algae were cultured under moderate light, only two metabolites mirrored the positive correlation between growth rate and total metabolite concentration observed for total metabolite levels, BCA (*P* = 0.027, *r* = 0.559) and DBA marginally (*P* = 0.070, *r* = 0.489). The correlation between the major metabolite bromoform tended positive (*P* = 0.204, *r* = 0.348) whereas dibromoacrylic acid tended negative (*P* = 0.273, *r* = −0.301).

ANOVAs testing variation in metabolite concentrations between the two light levels showed that light had a significant effect in most univariate tests. Algae cultured under moderate light had higher growth rates (*F*_1,14_ = 97.96, *P*<0.001) and higher total levels of metabolites (*F*_1,14_ = 21.92, *P*<0.001), as well as higher levels of bromoform (*F*_1,14_ = 21.00, *P*<0.001), CHBr_2_Cl (*F*_1,14_ = 6.77, *P* = 0.012) and DBA (*F*_1,14_ = 5.15, *P* = 0.04). Dibromoacrylic acid had lower levels in moderate compared to low light (*F*_1,14_ = 16.96, *P*<0.001). BCA did not differ between treatments. There were no significant interactions between light level and families for any analysis, which means that the fastest growing individuals under higher light also had the highest metabolite concentrations.

### Cellular Trends in Resource Allocation

**Figure 3:**
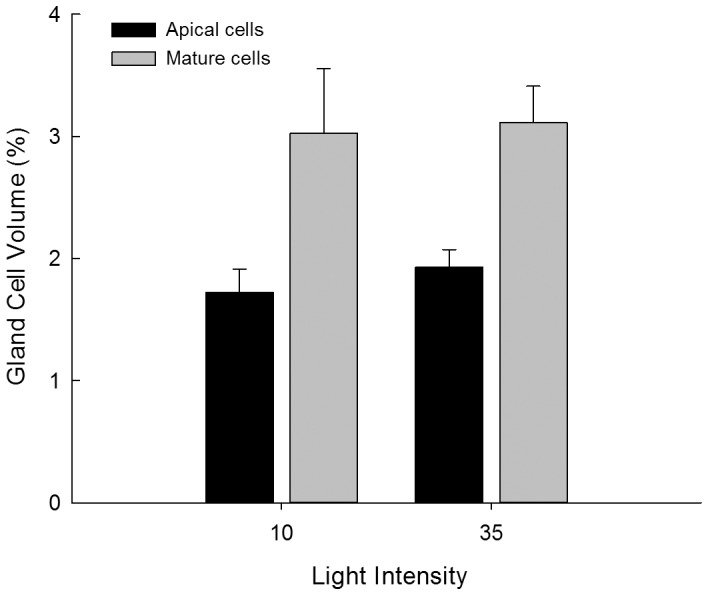
Empirical means of the percent volume (+1 SE) of the algal filament that is occupied by gland cells for apical and mature cells along the axis. Results represent the combined treatments for algae cultured under 10 µmol photons m^−2^ s^−1^ and 35 µmol photons m^−2^ s^−1^. *N* = 10 individuals per treatment.

The relative volume of the cell tier occupied by gland cells was significantly smaller in apical regions than in older (less distal) growth regions (Fig. 3, Table 2). There was no difference in mean values between light treatments and no interaction between light and growth region (Table 2). There was, however, a significant effect of family, indicating that variation in the gland cell volume among individuals tended to be more influential than variation due to light intensity. There was no gland cell present in the apical cell itself. The first cell tier that contained a gland cell was typically three to four cells below the apical cell, which did not differ between the light treatments (2-sample *T*-test, *P* = 0.198, *N* = 11, 13).

**Table 2:** Resource allocation across the growth axis.

Source	df	MS	*F*	*P*
Light	1	0.095	1.43	0.248
Region	2	1.672	25.14	0.001
Light x Region	2	0.002	0.03	0.104
Family	6	0.195	2.93	0.036
Error	12	0.066		

ANOVA results for the percentage of cell tier volume that is occupied by gland cells in apical and mature regions for algae cultured under low (10 µE) and moderate (35 µE) light. Data were log-transformed prior to analysis.

### Parameterisation and Validation of the Model

**Figure 4:**
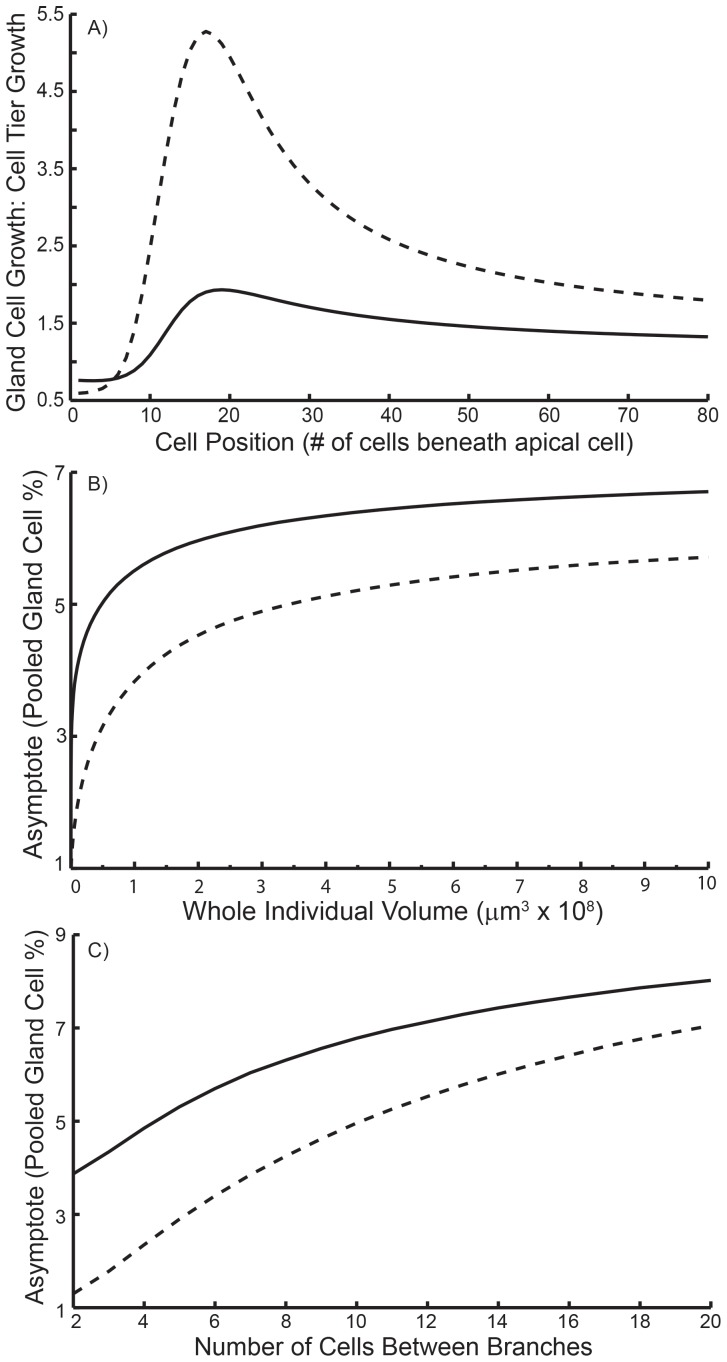
Key model simulations distinguishing low light (10 µE, dashed lines) and moderate light (35 µE, solid lines). (A) Ontogenetic trajectory of the relative investment in defence along a filament. (B) Pooled gland cell volume versus whole individual volume based on mean branch frequency (low = 16 and moderate = 11 cells per branch). The pooled gland cell volume (%) indicates the overall level of defence. (C) Pooled gland cell volume as a percentage of whole individual volume related to branch frequency. Higher branch frequency (left) leaders to lower overall levels of defence in an individual. Note that the relative difference between light levels decreases with decreased branch frequency (left to right).

The model growth functions (eqn. 1 and eqn. 2) were parameterised with the volume of cell tiers and the associated gland cells as a function of distance or position below the apex (which also indicates relative cell age). The two light treatments (10 and 35 µE) were maintained throughout as separate models. The density function parameters, *α* and *β,* in the two growth functions were adjusted so that gland cell and cell tier growth mimicked data with less than 1% error between real and simulated size development (see Table 1 for parameter values). The relationship between gland cell and cell tier growth as a function of cell position for the two different light levels is shown in Figure 4A. Independent of light level, gland cell growth is initially lower than cell tier growth (<1 on y-axis), a relationship that reverses as the cells age and are further removed from the apical section (i.e. >20 cells, Fig. 4A). The pattern is most abrupt under low light conditions (dashed line), notably with a marginally lower investment in defence in the apex compared to moderate light individuals (solid line) (Fig. 4A). However, the influence of the abrupt peak in the relative growth of gland cells from cell tier position 10 through to 20 (Fig. 4A, for both low and moderate light) can only be evaluated when the frequency of each cell tier position is calculated by summing across multiple branches for each individual (following section).

### Scaling the Model to Whole Individual

By incorporating cell division and branch frequency into the model we were able to monitor pooled (whole individual) changes in chemical defence as an individual increases in size (Fig. 4B). The first branch frequency included were 16 and 11 cells per branch corresponding to mean values for algae grown under 10 µE and 35 µE, respectively. The defence level (whole gland cell volume) always asymptotes as the algae grow larger, independent of light level. These asymptotes are reached when there is a steady state with respect to the proportion of different types of cells (i.e. the relative proportion of apical through to mature cells no longer changes beyond a certain size). In the case of *A. armata*, there is a clear positive relationship between size and the proportion of the gland cells in the cell tier up until the asymptote for both light levels (Fig. 4B). This is explained by the smaller proportion of gland cells in apical cells which constitute a larger fraction of an alga early in development. However, as the proportion of mature cells increases during growth, so does the proportion of the pooled gland cell volume of the whole individual (Fig. 4B). We therefore predict that large individuals cultured under higher light will consistently have higher levels of defence than similar sized individuals cultured under low light. This prediction appears to also hold for the empirical data if we project the low and moderate light correlations to intersect on the x-axis (Fig. 2B). These size-based trends are independent of any cost of defence, although the magnitude of the cost will affect the asymptote and therefore the whole individual level of defence.

Modelling the range of possible branching levels for the two light levels shows that the asymptote for the percent volume of gland cells in an individual is a negative function of branch frequency (frequency is the inverse of the number of cells between branches, Fig. 4C). The increased proportion of apical cells during increased branching explains this pattern (fewer cells per branch), which lowers the overall level of defence (pooled gland cell volume) during steady state. The relationship tends to be stronger under low light, since these algae have slightly smaller gland cells in the apical region than algae grown under moderate light (see Fig. 3). We also find that under moderate light the asymptote drops rapidly when there are less than 8–10 cells per branch, which is similar to the mean of the empirical data (i.e. *b* = 11). The equivalent is between 14–16 cells per branch for algae grown under low light, which is modelled by *b* = 16 (see Table 1).

By removing the cost of gland cell production from eqn. 2, the model was also used to estimate the cost of defence for both light treatments, based on the sum of cell tier growth with and without size dependency to gland cells in the equation. It predicts that algae grown under moderate light (35 µE) have reduced growth of individuals that reaches a high of ∼7% for very small individuals comprised of 10–40 cell tiers. This cost subsequently decreases to around 3% after the overall level of defence asymptotes and individuals are larger in size. The higher cost early in development is due to the relatively large size of gland cells when they are first formed (gland cells are first present 4 to 8 cell tiers beneath the apical cell). The equivalent costs for algae growing under low light (10 µE) are lower, at ∼3% and 2%, respectively for small and larger individuals. The difference in magnitude of costs between the two light treatments also reflects a greater opportunity cost for individuals growing in moderate light compared to slower growing individuals under low light.

### Interaction between Branching and Cell Division Rates

In the previous section we demonstrated that a higher frequency of branching can lead to larger individuals with a lower overall level of defence, the reverse of what was found in the empirical data (which was higher levels of defence with increased light). However, there was also a significant difference between cell division rates of *A. armata* grown under low light (10 µE, mean of 1.3 cells per day ±0.24 SD) and moderate light (35 µE, mean of 2.5 cells per day ±0.59 SD) (*F*_1,5_ = 89.05, *P*<0.001), and significant variation between families, ranging from 1–4 cells per day (*F*_5,36_ = 7.89, *P*<0.001). A marginal interaction between light and family (*F*_5,36_ = 2.22, *P* = 0.073) suggests that variation in cell division rate between individuals will be greater for *A. armata* cultured under moderate light. These data allowed us to introduce time into the model, as an increase in light leads to higher cell division rates with greater variance between families.

**Figure 5:**
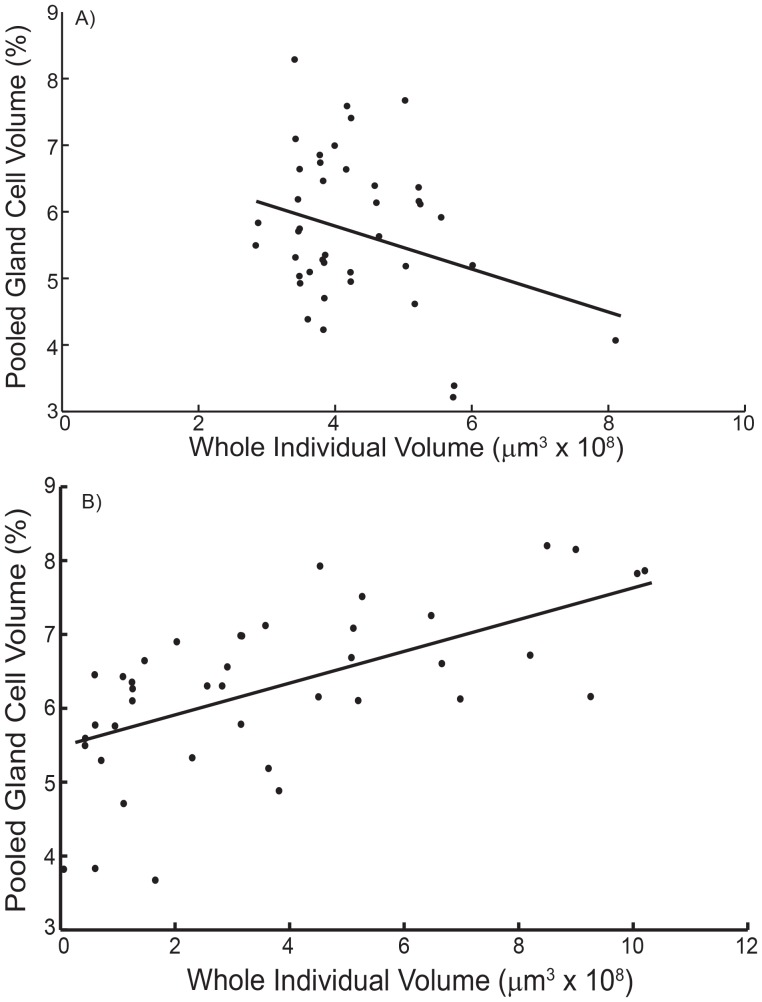
Model simulations of growth (whole individual – total volume) and defence levels (whole individual – pooled gland cell volume) under low (A) and moderate (B) light (n = 40 random individuals). (A) Under low light (10 µE) a negative correlation across individuals occurs with decreasing pooled gland cell volume in larger (more frequently branched) individuals. (B) Under moderate light (35 µE) a positive correlation is driven by greater variation in cell division between individuals producing individuals of different sizes, independent of branch frequency. Significant correlations are shown (*P*<0.05).

A negative correlation between growth and defence level under low light can be explained by differences in branch frequency between individuals (Fig. 5A, *P* = 0.048, *r* = 0.310). Branch frequency is the main driver of growth patterns under these low light conditions, as the variance in cell division rates among families was small. Therefore larger, more frequently branched, individuals have a lower asymptote for the pooled gland cell volume of the whole individual. A positive correlation between growth and defence levels at moderate light can be explained by the interactive effects of variation in cell division and branch frequency on growth pattern (Fig. 5B, *P*<0.001, *r* = 0.520). Under moderate light there tends to be more frequent branching (see Fig. 5A) but more importantly there is a higher variance in cell division rates. By altering the cell division rates, the model can also be used to generate individuals of vastly different sizes as before with branch frequency. This produces a different result, because it creates a population of individuals with different investment histories, based on the relative frequencies of apical and mature cells (from Fig. 4A). In this scenario the majority of the largest individuals still contain the highest overall levels of defence and drive the positive correlation (Fig. 5B).

## Discussion

The filamentous tetrasporophyte stage of the chemically defended red alga *Asparagopsis armata* has a simple axial growth form, comprised of tiers of somatic and defence (gland) cells that are repeated throughout the entire individual. It also has a simple growth pattern, with cell division from a single apical cell and regular branching events in the apical region. These features enabled us to develop a differential equations model for the growth of somatic and gland cells that described the outcome of a trade-off in the allocation of resources for the development of each cell tier, which was then summed to the scale of whole individuals. The most important finding was that contrasting outcomes for growth and chemical defence could be generated simply by manipulating growth patterns by using different combinations of branch frequency and cell division rates derived from empirical data. By treating investment in somatic cells and gland cells as two separate but related growth functions, we were able to formally demonstrate that seemingly small differences in branch frequency or cell division rates can lead to complex – and divergent – outcomes. Our results demonstrate that the relationship between one resource (light) and the concentration of constitutive chemical defences can be complex for organisms with simple body plans. Therefore using whole-individual correlations as a means to assess potential trade-offs between growth and defence in seaweeds (e.g. the cost of chemical defences) is fraught by the complexity of interactions likely operating at multiple independent scales, from the biochemistry of light energy harvesting, storage and use within cells, through to the competition for shared resources between neighbouring cells throughout ontogeny.

By using a modelling approach to investigate the relationships between growth patterns and the concentrations of constitutive chemical defences under hypothetical scenarios, we were able to provide some unique insights that can complement the reductionist approaches to quantifying the effects of light availability on specific biochemicals and/or growth. The model helped us to interpret contrasting and weak relationships between light availability, growth and concentrations of chemical defence in the empirical data by enabling the localised trade-offs at each cell tier to be expressed as the sum for a whole individual. For example, positive correlations between whole individual size and defence are predicted because somatic cell growth asymptotes before gland (defence) cell growth. However, growth asymptotes were also determined by branch frequency, because more branching increases the proportion of young to older cells in an individual by creating new growth axes. This means that if individuals of different sizes but the same age result from differences in branch frequency rather than cell division (as was the case under strong light limitation), then fast-growing, highly-branched individuals have relatively more young cells compared to slow growing individuals when they are small. In this scenario a negative correlation between size and defence is expected, because fast-growing individuals have proportionately more cells with lower levels of defence (smaller gland cells) than slow-growing individuals. It is notable that these results were irrespective of resource availability.

Complex interactions between resource levels, ontogeny, measures of performance such as growth, and concentrations of defensive metabolites have prevented consistent and predictable empirical outcomes for predicted trade-offs underlying the allocation of resources to chemical defence [3], [5], [7], [8]. The focus for terrestrial plants in the recent literature has notably switched from demonstrations of cost of chemical defence to analyses of how costs are managed by individuals throughout ontogeny, including when trade-offs should be expressed or measurable [8], [42]. However, the lack of clear trade-offs between growth and defence continues to remain a caveat in the interpretation of patterns of defence in seaweeds (e.g. [43], [44]), even though intrinsic features of seaweeds (such as high growth rates and simple body plans with complex life cycles) should make them useful to validate the plant defence theories in a different system. Our model explains the ontogeny of allocation to growth and defence in a simple filamentous alga but it also allowed us to identify constraints in empirical investigations of the cost of constitutive seaweed chemical defence. Both the model and empirical data demonstrate an overarching influence of growth patterns on any potential trade-off between growth and constitutive chemical defence in seaweeds, such that evidence for cost would be concealed very early in development. This outcome was independent of resource limitation, and driven by the highly plastic growth patterns of *Asparagopsis armata*.

The model demonstrated that correlative analyses of cost in seaweeds will be obscured in larger individuals because of the increase in the relative amount of older to younger cells, leading to a higher asymptote for whole-individual defence because of the corresponding increase in defence investment as cells age. The differences in branching and cell division rates for *A. armata* under low versus moderate light provided sufficient variation in individual size to explain the observed differences in whole individual defence. This plastic response to environmental stimuli could be integral to minimising trade-offs between growth and defence in fast growing seaweeds, modifying growth patterns by altering branching and cell division with continuous environmental feedback and dampening trade-offs at critical points during development. Our results provide additional support for so-called “transient” costs of defence - costs that are only detectable at certain points in ontogeny – which are expected to minimise opportunity costs for chemically-defended plants [45]. However, in our system there was not an abrupt shift in allocation to defence, rather a slower rate of investment in defence compared to somatic growth. Both the empirical and modelled data highlight that the single most important driver of the growth and defence of an individual is the different rate of investment in allocation to defence between young, actively growing cells and older cells. This pattern is the quintessential prediction of the growth-differentiation balance hypothesis (GDBH, [1]).

If the dampening of the cost of constitutive chemical defence in actively growing regions is an indirect outcome of increased branch frequency, resulting from increased light availability, then these trends conform with the GDBH which promotes growth prior to defence. Furthermore, the absence of gland cells in the apical cell of *A. armata* implies little or no trade-off between defence and cell division, meaning that cell division rates are unlikely constrained by resource allocation. An analogous strategy for terrestrial plants to minimise opportunity costs in important growth regions is to delay production of the more costly metabolites. For example, specific metabolites are absent in early stages of seedling development [9]. We also found conflicting trends for specific metabolites in *A. armata*, with different degrees and strengths of correlations between metabolite concentration and growth rates dependent on light level, which is similar to the variability between furanone analogues in the red seaweed *Delisea pulchra* [12]. If quantitative chemical data can be supported with an independent measure such as cellular data [12], [40], then this provides an additional means to interpret contrasting correlations between metabolite concentrations and growth.

Changes in allocation to production of secondary metabolites with respect to growth stage are important to resource-based defence models, but can be difficult to partition for many higher plants because of ontogeny and the translocation of resources throughout individuals [1], [8], [9]. Complexities of ontogeny and body plan are reduced in seaweeds, particularly for filamentous algae in which growth is from a single apical cell and little or no differentiation occurs prior to reproduction. By treating the growth of somatic and gland cells as distinct but related functions, we were able to monitor the ontogeny of allocation to defence and sum any costs of constitutive chemical defences for individuals with different growth histories. The model showed that the consequence of allocation costs in *A. armata* (a 3–8% reduction in growth of young somatic cells) was negligible when summed together for large, highly branched and thus older individuals. Therefore opportunity costs, of up to 10% reduction in performance, will only be measurable at very early developmental stages (e.g. individuals comprised of between 10 and 20 cells), consistent with the recent emphasis on investigating costs in plant seedlings and early growth stages of terrestrial plants [9], [45]. However, targeting early growth stages does not guarantee a trade-off, as positive correlations have also been found between growth and defence for young plants or seedlings [45], [46]. One explanation is that systemic (whole-individual) trade-offs are not tightly coupled in young plants, similar to seaweeds, perhaps because source-sink relationships are under-developed or biased at particular ontogenetic stages. It is notable that our observed increase in constitutive chemical defences of seaweeds across ontogeny, as predicted by the GDBH, mirrors the pattern for herbs and is distinct from woody plants [42]. One weakness in our approach is that we have not addressed the potential flow on effects of allocation constraints to reproductive output; however, reproduction in these seaweeds is yet another example of a localised event as spores are formed by a single pericentral cell in the cell tier. Size or total cell number is therefore likely to be the most relevant proxy for reproductive fitness in *A. armata*, but this remains to be tested. Another potential limitation is that excess light energy may be captured and stored as floridean starch granules, which could fracture any direct relationship between growth and concentration of chemical defences in *Asparagopsis*; however, these highly refractive bodies were not apparent in the study and were typically only observed in older cultures [30].

We have presented a model which formalises the difference between cellular allocation costs and growth allocation costs at the scale of whole individuals. Most importantly, the model demonstrates that if allocation costs exist in these filamentous algae, then they can be dampened or constrained by morphology, and that positive correlations between growth and defence are predicted in many circumstances. Our empirical and modelled data tend to reflect the predictions of the GDBH, as chemical defence has a lower rate of increase than somatic growth across the growth axis in all but the first 5–10 cells, and cell division is not limited by allocation to defence. Allocation costs in seaweeds may therefore only be detected at very early life stages, after which trade-offs between growth and defence will be concealed by innate growth patterns. However, one caveat with extrapolating the model to all seaweeds is that allocation costs may not be localised for species which have rudimentary translocation systems (e.g. kelps and other brown algae [27], [43]) or those with siphonous structure [47]. Regardless the model highlights a need to understand the ontogeny of the individual before the consequences of the costs of constitutive chemical defence in seaweeds can be rationalised. Factors that influence individual size and ontogenetic state, specifically plasticity in branching and cell division rates in *Asparagopsis* ([34]; this study), appear to be more important to the level of defence level of an individual than any localised trade-off in resource allocation between somatic cells and defence cells. The ability to respond plastically to resource variation may dampen trade-offs between growth and defence, and could also contribute to the extraordinarily high biomass productivity of the chemically defended *A. armata*, >80g dry weight m^−2^ day^−1^, one of the highest reported for any photosynthetic organism [48].
